# Editorial: Genomics-driven advances in crop productivity and stress resilience

**DOI:** 10.3389/fpls.2025.1645714

**Published:** 2025-07-11

**Authors:** Parimalan Rangan, Robert Henry, Ambika Baldev Gaikwad

**Affiliations:** ^1^ Division of Genomic Resources, ICAR-National Bureau of Plant Genetic Resources, New Delhi, India; ^2^ Queensland Alliance for Agriculture and Food Innovation, The University of Queensland, St Lucia, QLD, Australia

**Keywords:** genomics, germplasm (genetic) resources, climate change, crop wild relatives (CWRs), crop productivity, stress resilience

Scaling up crop productivity in response to climate change is critical to sustainably feeding the ever-growing population. The rate of genetic gain being achieved in recent decades needs to be augmented to meet this demand (van Dijk et al., 2021; Hunter et al., 2017). Genomic selection and gene editing strategies for *de novo* domestication, breaking linkage drag, and overcoming genetic incompatibility barriers are genomics-driven tools that have been demonstrated to enhance crop productivity and stress resilience. Strategies such as landscape genomics, which also consider environmental variables, increase the potential utilization of genebank collections, including crop wild relatives (CWRs) and ‘exotic genetic libraries’ through identification of appropriate accessions for utilization (Bohra et al., 2022; Campbell et al., 2025; Shrestha et al., 2025). Genomics tools also help gain novel insights into the genetic and epigenetic mechanisms associated with stress resilience traits and the factors to target for enhanced crop productivity (Bailey-Serres et al., 2019; Gupta, 2025; Lohani et al., 2025; Miryeganeh, 2025). Understanding the role of the interplay of various components such as transposable elements (Tossolini et al., 2025) secondary metabolites (Khan, 2025) and small peptides (Xiao et al., 2025) in influencing stress resilience will help us devise strategies to utilize genomics tools to improve crop plants and crop diversification (Wang et al., 2025).

Here, Krishnan et al. demonstrated the applicability of genomics-assisted tools in a wide-hybridization program involving a heat-tolerant diploid wild wheat *Aegilops* sp*eltoides* accession and a *Triticum durum* accession to derive a backcross introgression line (BIL) population. Using this population for marker-assisted selection (MAS), the research group identified 30 QTLs for heat stress tolerance using molecular breeding and genotyping-by-sequencing (GBS) approaches involving both SSR and SNP markers to map the QTLs. Linkage disequilibrium (LD) decay values calculated using Tassel v5.0 helped the authors target 21 candidate genes associated with heat stress tolerance. The most prominent targets based on functional annotation were: cytochrome P450, ABC transporters, E3 ubiquitin-protein ligase, Alcohol dehydrogenase, the F-box family, and the MYB family. Similarly, in grapevine berries, Heinekamp et al. used a mapping population (Calardis Musque x Villard Blanc) and important cultivars such as ‘Calardis Blanc’ (a sunburn-resilient cultivar), totaling a population of 150 for phenotypic evaluation for sunburn resilience in addition to fungal resistance in grapevine cultivars. Using the composite interval mapping (CIM) approach with five years of phenotypic data, along with a genetic map of grapevines, they successfully identified two QTLs that explain approximately 40% of the phenotypic variance and were found to be on chromosomes 10 and 11. With a greater number of heat waves in recent years, climate-change-adapted cultivars that are resistant to fungus and are sunburn resilient are required for sustained viticulture to ensure high yields and wine quality.

Using a population genomics approach, Zhang et al. demonstrated the influence of low temperature in exerting selection pressure on various plant traits during the adaptation of a *Kandelia obovata* population. To accomplish this, the team introduced a population of *K. obovata* from Zhangzhou (ZZ) to two different locations, Quanzhou (QZ, 2003) and Wenzhou (WZ, 2005). Morphological differences were observed at the two sites. To understand the underlying genetics of this variation, the researchers used a whole-genome resequencing approach and identified the SNPs that varied between the original habitat and the introduced habitat. The positive selection for genes associated with the SNPs (from the northern province WZ) was analyzed to reveal candidate genes linked especially to cold tolerance and glutathione metabolism traits. Analysis of their promoter sequences (extracted up to 2 kbp) showed enrichment of elements primarily linked to stress tolerance, such as stress-, low temperature-, wound-, and abscisic acid (ABA)-responsive elements. 

To achieve drought stress resilience in tropical maize and lettuce, de Pontes et al. and Medina-Lozano et al. utilized different genomic tools to identify candidate genes in these crops. Using 360 maize inbred lines, SNP-array genotype data from an Affymetrix platform and GBS approaches, the researchers identified drought-associated SNPs and in turn the underlying candidate genes through genome-wide association studies (GWAS). The candidate genes were found to be associated with key pathways such as ethylene biosynthesis, jasmonic acid biosynthesis, gibberellin biosynthesis, and ABA biosynthesis, along with specific protein families such as the TPR, PPR, PR, and MYB families. They were also found to be associated with genes such as shoot gravitropism 5, and circadian clock genes. These findings will contribute to improving maize cultivars tailored with drought resilience for sustained yield improvement. For lettuce, the authors used an RNA-seq approach to study differential expression between the lettuce cultivar ‘Romired’ and the wild lettuce relative *Lactuca homblei*. The latter is known to significantly overexpress the anthocyanins during drought. Through this study, the authors identified 36 genes, with approximately 50% of them linked to the phenylpropanoid-flavonoid pathway. The other genes were annotated as being associated with stomatal closure, phospholipases, and transcription factors such as MYB, NAC56, PRA1, HSC70, and ZAT1. This provides insight into the regulation of the drought-mediated anthocyanin pathway for use in imparting drought resilience from the wild species to cultivated lettuce.

In order to promote the molecular breeding and marker-assisted development of novel cultivars of the ornamental plant *Cymbidium ensifolium*, Shen et al. utilized the double-digest restriction site-assisted DNA sequencing (ddRAD-seq) technique to sequence 50 commercially available cultivars and identified approximately 1.2 million high-quality SNPs. From these SNPs, competitive allele-specific PCR (KASP) primers were designed and used to screen the cultivars and found that 11 of the final 28 KASP markers are sufficient to distinguish the 83 cultivars tested.

In an effort to generate a single circular mitochondrial genome for the decaploid species *Camellia hainanica* (octaploids are also available), Zhang et al. used Illumina short-read and Nanopore long-read sequencing technologies to generate raw data. After initial filters of the raw data using fastp v0.20.0 and filtlong v0.2.1, the data were mapped against plant mitochondrial core genes using Minimap2 to extract the mitochondrial sequences for assembly using SPAdes v3.15.4, yielding a single circular mitochondrial genome of 902,617 bp in length. The mitochondrial genome was annotated for genes (protein-coding and non-coding) and SSR markers.

The value of conserving germplasm is realized when it is practically utilized in a breeding program to develop cultivars introgressed with key desirable traits from germplasm that would otherwise be difficult to introduce. He et al. evaluated 361 soybean germplasm accessions, comprising six maturity groups, for variability in 100-seed weight (100SW) and seed oil content (SOC). Using a restricted two-stage multi-locus genome-wide association study (RTM-GWAS) approach, LD blocks comprising 230 and 299 alleles, for 100SW and SOC, respectively, were identified. Gene annotation studies revealed 87 and 132 candidate genes for the 100SW and SOC traits, respectively. Promising 100SW genes included vacuolar proton ATPase A3 and clathrin adaptor complexes. The most promising gene for SOC was identified to be a HAD superfamily phosphatase gene. Genomic selection models using data from the 361 soybean germplasm helped predict the recombination potential of the two studied traits, 100SW (up to 30.43 g) and SOC (up to 27.73%). A model based on priority traits can be chosen for implementation in a breeding program to improve oil yield. Zhernova et al. reviewed the genetic markers available for the improvement of flax (*Linum usitatissimum*), which is widely cultivated for its oil and fiber. Their review underscores the markers identified for various traits of interest including biotic and abiotic stress tolerance. This compiled information will be ready-to-use for marker-assisted flax improvement.

In conclusion, the enormous growth in data generation and computational power that drives accelerated crop improvement has transformed genomics applications for innovations. Notable advances include the move from single-reference to pan-genome references (Ruperao et al., 2025), understanding the synergy of the microbiome in crop improvement particularly in terms of yield and stress resilience (Xu et al., 2025; Ge and Wang, 2025), identifying susceptibility genes (Baruah et al., 2025), and *de novo* domestication and rational redomestication (Wang et al., 2025). These advances will guide research in the coming decades as it addresses the challenges of sustainable agriculture. 
